# Why Is there a Limit to the Changes in Myofilament Ca^2+^-Sensitivity Associated with Myopathy Causing Mutations?

**DOI:** 10.3389/fphys.2016.00415

**Published:** 2016-09-26

**Authors:** Steven B. Marston

**Affiliations:** National Heart & Lung Institute, Imperial College LondonLondon, UK

**Keywords:** muscle regulation, Ca^2+^-sensitivity, troponin C, HCM, DCM, myopathy, mutation

## Abstract

Mutations in striated muscle contractile proteins have been found to be the cause of a number of inherited muscle diseases; in most cases the mechanism proposed for causing the disease is derangement of the thin filament-based Ca^2+^-regulatory system of the muscle. When considering the results of experiments reported over the last 15 years, one feature has been frequently noted, but rarely discussed: the magnitude of changes in myofilament Ca^2+^-sensitivity due to myopathy-causing mutations in skeletal or heart muscle seems to be always in the range 1.5–3x EC_50_. Such consistency suggests it may be related to a fundamental property of muscle regulation; in this article we will investigate whether this observation is true and consider why this should be so. A literature search found 71 independent measurements of HCM mutation-induced change of EC_50_ ranging from 1.15 to 3.8-fold with a mean of 1.87 ± 0.07 (sem). We also found 11 independent measurements of increased Ca^2+^-sensitivity due to mutations in skeletal muscle proteins ranging from 1.19 to 2.7-fold with a mean of 2.00 ± 0.16. Investigation of dilated cardiomyopathy-related mutations found 42 independent determinations with a range of EC_50_ wt/mutant from 0.3 to 2.3. In addition we found 14 measurements of Ca^2+^-sensitivity changes due skeletal muscle myopathy mutations ranging from 0.39 to 0.63. Thus, our extensive literature search, although not necessarily complete, found that, indeed, the changes in myofilament Ca^2+^-sensitivity due to disease-causing mutations have a bimodal distribution and that the overall changes in Ca^2+^-sensitivity are quite small and do not extend beyond a three-fold increase or decrease in Ca^2+^-sensitivity. We discuss two mechanism that are not necessarily mutually exclusive. Firstly, it could be that the limit is set by the capabilities of the excitation-contraction machinery that supplies activating Ca^2+^ and that striated muscle cannot work in a way compatible with life outside these limits; or it may be due to a fundamental property of the troponin system and the permitted conformational transitions compatible with efficient regulation.

Mutations in striated muscle contractile proteins have been found to be the cause of a number of inherited muscle diseases; in most cases the mechanism proposed for causing the disease is derangement of the thin filament-based Ca^2+^-regulatory system of the muscle. Hypertrophic cardiomyopathy and hypercontractile diseases of skeletal muscle, such as distal arthrogryposis and “stiff child syndrome,” have been linked to a higher myofilament Ca^2+^-sensitivity (Marston, 2011; Donkervoort et al., 2015). In contrast dilated cardiomyopathy mutations are commonly, but not exclusively, linked to decreased Ca^2+^-sensitivity. Mutations in contractile proteins that are linked to nemaline myopathy and related skeletal muscle myopathies have also been found to be associated with reduced Ca^2+^ sensitivity (Marttila et al., 2012, 2014). The causative connection between myofilament Ca^2+^-sensitivity and muscle dysfunction is a field of intensive research that is too complex to consider in this account. However, when considering the results of such experiments reported over the last 15 years, one feature has been frequently noted, but rarely discussed. The magnitude of changes in myofilament Ca^2+^-sensitivity due to myopathy-causing mutations in skeletal or heart muscle seems to be always in the range 1.5–3x EC_50_. Such consistency suggests it may be related to a fundamental property of muscle regulation; in this article we will investigate whether this observation is true and consider why this should be so.

Most investigations have found increased Ca^2+^-sensitivity in muscle with hypertrophic cardiomyopathy (HCM) and restrictive cardiomyopathy (RCM)-causing mutations. Our literature search found 71 independent measurements of the mutation-induced change of EC_50_ ranging from 1.15 to 3.8-fold with a mean of 1.87 ± 0.07 (sem) (Table 1). We also found 11 independent measurements of increased Ca^2+^-sensitivity due to mutations in skeletal muscle proteins ranging from 1.19 to 2.7-fold with a mean of 2.00 ± 0.16 (Table 2).

**Table 1:** **Effect of HCM-associated mutations on myofilament Ca^2+^-sensitivity**.

**Gene name**	**Mutation**	**wt/mutant EC_50_ ratio**	**Measured in**	**References**
**HCM**				
ACTC	E99K	2.45	IVMA	Song et al., 2011
ACTC	E99K	1.24	IVMA (human)	Song et al., 2011
ACTC	E99K	1.89	IVMA	Papadaki et al., 2015
ACTC	E99K	1.3	Fibers TG	Song et al., 2011
ACTC	E99K	2.35	Myofibrils TG	Song et al., 2013
MYL2	R58Q	1.29	Fibers X	Szczesna-Cordary et al., 2004
MYL2	D166V	1.78	Fibers TG	Kerrick et al., 2009
MYL2	D166V	1.82	Fibers TG	Yuan et al., 2015
MYH7	R403Q	1.79	Human fibers	Sequeira et al., 2013
MYH7	R403Q	1.41	Fibers TG	Blanchard et al., 1999
MYH7	R453C	1.99	Human fibers	Palmer et al., 2004
MYBPC3	Cat R820W	2.01	IVMA	Messer et al., 2016a
MYBPC3	“KI”	1.35	Fibers TG	Fraysse et al., 2012
MYBPC3	E258K	1.80	Human fibers	Sequeira et al., 2013
TNNC1	A8V	2.51	Fibers TG	Martins et al., 2015
TNNC1	A8V	2.3	Fibers X	Pinto et al., 2009
TNNC1	L29Q	1.26	Fibers X 2.3 μm	Li et al., 2013
TNNC1	L29Q	1.17	Fibers X 1.9 μm	Li et al., 2013
TNNC1	L29Q	2.1	IVMA	Schmidtmann et al., 2005
TNNC1	A31S	1.48	Fibers X	Parvatiyar et al., 2012
TNNC1	A31S	2.75	ATPase	Parvatiyar et al., 2012
TNNC1	D145E	1.74	Fibers X	Pinto et al., 2009
TNNC1	C84Y	1.86	Fibers X	Pinto et al., 2009
TNNI3	R21C	2.16	Fibers X	Gomes et al., 2005a
TNNI3	L144Q	2.04	Fibers X	Gomes et al., 2005b
TNNI3	R145G	3.63	ATPase	Elliott et al., 2000
TNNI3	R145G	2.09	ATPase	Takahashi-Yanaga et al., 2001
TNNI3	R145G	1.82	IVMA	Brunet et al., 2014
TNNI3	R145G	1.41	IVMA	Deng et al., 2001
TNNI3	R145G	1.35	Fibers X	Lang et al., 2002
TNNI3	R145G	1.15	Fibers TG	Krüger et al., 2005
TNNI3	R145Q	1.41	Fibers X	Takahashi-Yanaga et al., 2001
TNNI3	R145Q	1.70	ATPase	Takahashi-Yanaga et al., 2001
TNNI3	R145W	2.45	Fibers X	Gomes et al., 2005b
TNNI3	R145W	1.15	Human fibers	Sequeira et al., 2013
TNNI3	R162W	1.28	ATPase	Takahashi-Yanaga et al., 2001
TNNI3	A171T	1.38	Fibers X	Gomes et al., 2005b
TNNI3	K178E	2.95	Fibers X	Gomes et al., 2005b
TNNI3	ΔK182	1.51	ATPase	Takahashi-Yanaga et al., 2001
TNNI3	ΔK183	3.8	IVMA	Köhler et al., 2003
TNNI3	R192H	2.29	Fibers X	Gomes et al., 2005b
TNNI3	G203S	3.02	IVMA	Köhler et al., 2003
**HCM**				
TNNI3	K206Q	2.51	IVMA	Köhler et al., 2003
TNNI3	K206Q	1.51	ATPase	Takahashi-Yanaga et al., 2001
TNNI3	K206I	1.81	ATPase	Warren et al., 2015
TNNT2	TnTΔ14	2.51	Fibers X	Gafurov et al., 2004
TNNT2	TnTdel	2.69	ATPase	Redwood et al., 2000
TNNT2	I79N	1.41	Fibers X	Szczesna et al., 2000
TNNT2	I79N	2.04	Fibers TG	Baudenbacher et al., 2008
TNNT2	R92L	1.65	Fibers TG	Ford et al., 2012
TNNT2	R92Q	1.66	Fibers TG	Ford et al., 2012
TNNT2	R92Q	1.74	ATPase	Robinson et al., 2002
TNNT2	R92Q	1.94	IVMA	Robinson et al., 2002
TNNT2	F110I	2.34	Fibers TG	Szczesna et al., 2000
TNNT2	F110I	1.32	Fibers TG	Baudenbacher et al., 2008
TNNT2	ΔE160	1.41	Fibers TG	Lu et al., 2003
TNNT2	R278C	2.19	Fibers TG	Szczesna et al., 2000
TNNT2	K280N	1.64	IVMA	Messer et al., 2016b
TNNT2	K280N	1.26	IVMA (human Tn)	Messer et al., 2016b
TPM1	E62Q	1.21	ATPase	Chang et al., 2005
TPM1	A63V	1.91	Transfected cell	Michele et al., 1999
TPM1	A63V	1.99	ATPase	Heller et al., 2003
TPM1	K70T	1.58	Transfected cell	Michele et al., 1999
TPM1	K70T	2.13	ATPase	Heller et al., 2003
TPM1	D175N	1.23	IVMA	Bing et al., 2000
TPM1	E180G	1.30	IVMA	Bing et al., 2000
TPM1	E180G	1.63	IVMA	Papadaki et al., 2015
TPM1	E180G	1.44	Transfected cell	Michele et al., 1999
TPM1	E180G	2.75	ATPase	Chang et al., 2005
TPM1	L185R	2.51	ATPase	Chang et al., 2005
TPM1	I284V	1.50	Human fibers	Sequeira et al., 2013

*The criteria for inclusion in the table are (1) that a missense mutation has been convincingly linked to the myopathy phenotype and (2) that only direct Ca^2+^-sensitivity comparisons of mutant and “normal” are included. Seventy-one independent measurements of the HCM mutation-induced change of EC_50_ shown as EC_50_ WT/mutant. Values range from 1.15 to 3.8-fold with a mean of 1.87 ± 0.07 (sem). Shading indicates gene studied*.

*Gene names: ACTC, cardiac alpha actin; TNNI3, cardiac troponin I; TNNT2, cardiac troponin T (T3 isoform); TNNC2 cardiac troponin C; MYL2, ventricular regulatory myosin light chain; MYH7, beta myosin heavy chain; MYBPC3, cardiac myosin binding protein C; TPM1, alpha tropomyosin, Tpm1.1*.

*Measurement methods: IVMA, in vitro motility assay; Fibers TG, skinned fibers from transgenic or knock-in mouse heart; Myofibrils TG, single myofibrils from transgenic or knock-in mouse heart; Fibers X, skinned fibers with mutation protein exchanged in Human fibers, skinned fibers from human heat muscle; ATPase, reconstituted thin filament activation of myosin ATPase activity*.

**Table 2:** **Effect of skeletal muscle gain-of -function mutations on Ca^2+^-sensitivity shown as EC_50_ WT/mutant**.

**Gene name**	**Mutation**	**wt/mutant EC_50_ ratio**	**Measured in**	**References**
ACTA1	K326N	2.50	IVMA	Jain et al., 2012
TPM2	ΔK49	1.19	IVMA	Marston et al., 2013
TPM2	ΔE139	1.51	IVMA	Marston et al., 2013
TPM2	E181K	1.58	Human fibers	Ochala et al., 2012
TPM2	ΔK7 50%	2.00	IVMA	Mokbel et al., 2013
TPM2	ΔK7	2.70	Human fibers	Mokbel et al., 2013
TPM3	K168E	2.67	IVMA	Marston et al., 2013
TPM3	K168E 50%	1.85	IVMA	Marston et al., 2013
TPM3	ΔE224	1.34	Human fibers	Donkervoort et al., 2015
TPM3	ΔE224	2.2	IVMA	Donkervoort et al., 2015
TPM3	Δ218	2.5	IVMA	Donkervoort et al., 2015

*The mean change is 1.65± 0.16-fold (range 1.19–2.70)*.

GENE NAMES: ACTA1, skeletal muscle alpha actin; TPM2, beta tropomyosin, Tpm2.2; TPM3, Tpm3.12, “gamma tropomyosin.”

*Shading indicates gene studied*.

Dilated cardiomyopathy-causing mutations were initially found to decrease Ca^2+^-sensitivity but more recent studies have indicated the situation is more complex. DCM-linked mutations can both increase and decrease Ca^2+^-sensitivity depending on the individual mutations, moreover the direction of change can be different with a single mutation measured in different systems (Marston, 2011; Memo et al., 2013). This is illustrated in Table 3 where 42 independent determinations show a range of EC_50_ wt/mutant from 0.3 to 2.3. In addition we found 14 measurements of Ca^2+^-sensitivity changes due skeletal muscle myopathy mutations ranging from 0.39 to 0.63 (Table 4).

**Table 3:** **Effect of dilated cardiomyopathy linked mutations on Ca^2+^-sensitivity**.

**Gene name**	**Mutation**	**wt/mutant EC_50_ ratio**	**Measured in**	**References**
ACTC	E361G	1.05	IVMA	Song et al., 2010
ACTC	E361G skTn	0.30	IVMA	Song et al., 2010
TNNI3	K36Q	0.47	IVMA	Memo et al., 2013
TNNI3	K36Q	0.41	ATPase	Carballo et al., 2009
TNNI3	N185K	0.42	ATPase	Carballo et al., 2009
TNNT2	R131W	0.59	ATPase	Mirza et al., 2005
TNNT2	R131W	0.63	IVMA	Mirza et al., 2005
TNNT2	R134G	0.89	Fibers X	Hershberger et al., 2009
TNNT2	R141W	0.69	IVMA	Memo et al., 2013
TNNT2	R141W	0.80	ATPase	Mirza et al., 2005
TNNT2	R141W	0.89	Fibers X	Venkatraman et al., 2005
TNNT2	R151C	0.81	Fibers X	Hershberger et al., 2009
TNNT2	R159Q	0.83	Fibers X	Hershberger et al., 2009
TNNT2	R206L	0.35	IVMA	Mirza et al., 2005
TNNT2	R205L	0.34	ATPase	Mirza et al., 2005
TNNT2	R205L	0.68	Fibers X	Mirza et al., 2005
TNNT2	R205W	0.83	Fibers X	Hershberger et al., 2009
TNNT2	ΔK210 hetero	0.63	IVMA	Du et al., 2007
TNNT2	ΔK210	0.75	Fibers X	Venkatraman et al., 2005
TNNT2	ΔK210	0.45	IVMA	Du et al., 2007
TNNT2	ΔK210 recombinant	1.54	ATPase	Mirza et al., 2005
TNNT2	ΔK210 50%	0.46	IVMA	Mirza et al., 2005
TNNT2	D270N	0.65	IVMA	Mirza et al., 2005
TNNT2	D270N	0.64	ATPase	Mirza et al., 2005
TNNC1	Y5H	0.82	Fibers X	Pinto et al., 2011
TNNC1	D73N	0.55	ATPase	McConnell et al., 2015
TNNC1	D73N	0.59	Fibers X	McConnell et al., 2015
TNNC1	D145E	0.52	Fibers X	Pinto et al., 2011
TNNC1	I148V	0.91	Fibers X	Pinto et al., 2011
TNNC1	G159D	0.56	ATPase	Mirza et al., 2005
TNNC1	G159D	0.55	IVMA	Mirza et al., 2005
TNNC1	G159D	1.86	IVMA	Dyer et al., 2009
TNNC1	G159D skTn	0.56	IVMA	Dyer et al., 2009
TNNC1	G159D		Fibers X	Biesiadecki et al., 2007
TPM1	E40K	0.69	IVMA	Memo et al., 2013
TPM1	E40K baculovirus	0.38	IVMA	Memo et al., 2013
TPM1	E40K	0.64	ATPase	Chang et al., 2005
TPM1	E54K	0.58	ATPase	Mirza et al., 2005
TPM1	E54K	1.90	Ca binding	Robinson et al., 2007
TPM1	D230N baculovirus	2.30	IVMA	Memo et al., 2013
TPM1	D230N bacu+skTn	0.59	IVMA	Memo et al., 2013
TPM1	D230N Recombinant	0.54	ATPase	Lakdawala et al., 2010

*Forty-two independent measurements of the mutation-induced change of EC_50_ shown as EC_50_ WT/mutant*.

*Shading indicates gene studied*.

**Table 4:** **Skeletal myopathy mutations causing a loss of function**.

**Gene name**	**Mutation**	**wt/mutant EC_50_ ratio**	**Measured in**	**References**
TPM2	E117K	0.41	IVMA	Marttila et al., 2012
TPM2	Q147P	0.63	IVMA	Marttila et al., 2012
TPM3	L100M	0.52	IVMA	Marttila et al., 2012
TPM3	R167C	0.36	Myofibers	Ochala et al., 2012
TPM3	R167H	0.59	IVMA	Marston et al., 2013
TPM3	R167H 50%	0.58	IVMA	Marston et al., 2013
TPM3	R244G	0.46	IVMA	Marston et al., 2013
TPM3	R244G 50%	0.60	IVMA	Marston et al., 2013
TPM3	K169E	0.55	Myofibers	Yuen et al., 2015
TPM3	R245G	0.45	Myofibers	Yuen et al., 2015
TPM3	L100M	0.53	Myofibers	Yuen et al., 2015
TPM3	R168G	0.48	Myofibers	Yuen et al., 2015
TPM3	R168H	0.42	Myofibers	Yuen et al., 2015
TPM3	R167C	0.39	Myofibers	Yuen et al., 2015

*Fourteen independent measurements of the mutation-induced change of EC_50_ shown as EC_50_ WT/mutant. The mean change is 0.49 ± 0.02-fold (range 0.36–0.63)*.

*Shading indicates gene studied*.

Thus, our extensive literature search, although not necessarily complete, found that, indeed, the changes in myofilament Ca^2+^-sensitivity due to disease-causing mutations have a bimodal distribution and that the overall changes in Ca^2+^-sensitivity are quite small and do not extend beyond a 3–4-fold increase or decrease in Ca^2+^-sensitivity. Indeed when all the findings are plotted as a histogram one finds that increases in Ca^2+^-sensitivity on a log scale have an approximately normal distribution with mean increase in Ca^2+^-sensitivity (EC_50_ wt/mutant) of 1.86-fold (corresponding to ΔpCa_50_ = 0.255 ± 0.015), whilst the decreases in Ca^2+^ sensitivity have a mean EC_50_ wt/mutant of 0.54-fold (corresponding to ΔpCa_50_ of –0.286 ± 0.01; Figure 1A). It is also worth noting that this small Ca^2+^-sensitivity shift is observed independent of the measurement method Figure 1B compares the ΔpCa_50_ distribution measured by unloaded assays (actomyosin ATPase or *in vitro* motility) and by loaded assays (force measurements in skinned muscles, cell, and isolated myofibrils). The mean magnitude of the Ca^2+^-sensitivity change is about 20% less when measured in loaded assays.

**Figure 1:**
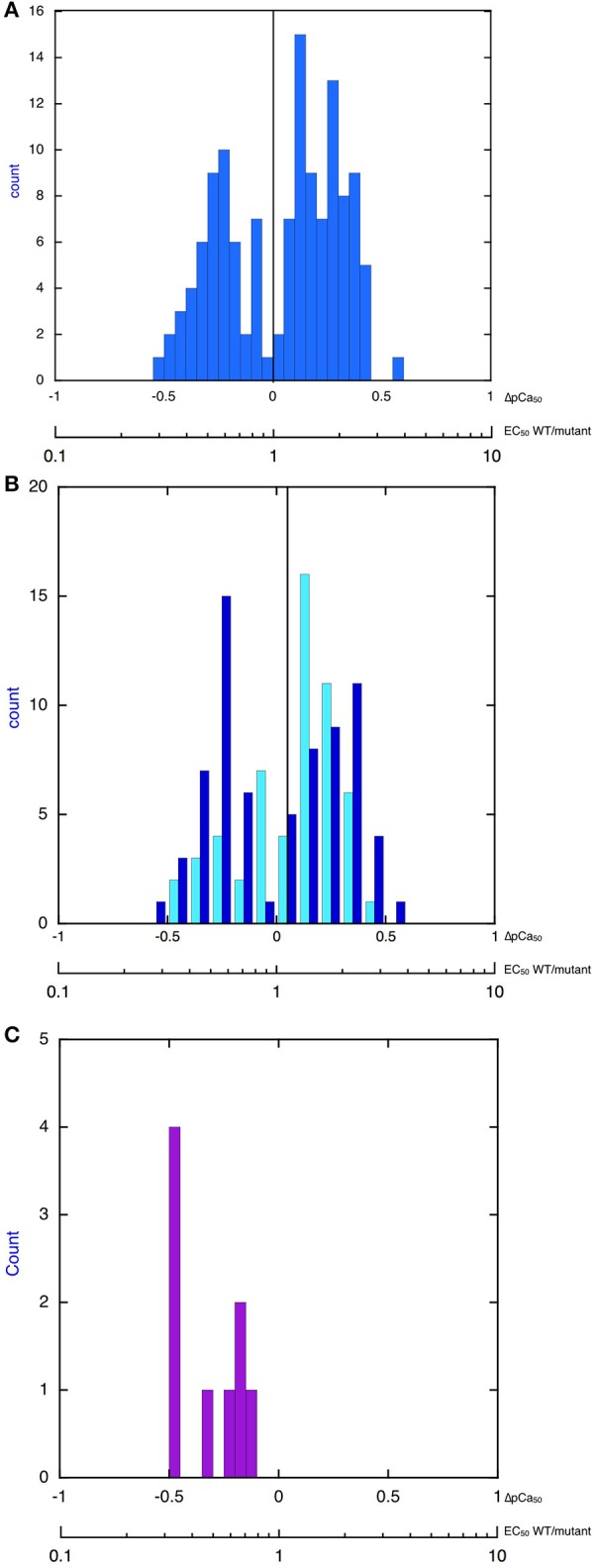
**Histograms showing distribution of the change in Ca^2+^-sensitivity due to mutations and phosphorylation**. The X-axis is pCa_50_(mutant-WT, ΔpCa_50_) or EC_50_ (WT/mutant), log scale. **(A)** All 149 values from Tables 1–4 are plotted. The plot is bimodal. Mean of decreased Ca^2+^-sensitivity (ΔpCa_50_ < 0) = –0.286 ± 0.016, Mean of increased Ca^2+^ sensitivity (ΔpCa_50_ > 0) = 0.255 ± 0.015. **(B)** Distribution of change in Ca^2+^-sensitivity is compared for loaded (pale blue) and unloaded (dark blue) assays of cardiac muscle regulation (data from Tables 1, 3). Unloaded assays are IVMA and ATPase, loaded assays are Fibers TG, Myofibrils TG, Fibers X, Human fibers, For decreased Ca^2+^ sensitivity mean unloaded ΔpCa50 is –0.27 ± 0.02 and mean loaded is –0.21 ± 0.03, *p* = 0.05. For increased Ca^2+^-sensitivity mean unloaded ΔpCa50 is 0.26 ± 0.02 and mean loaded is 0.021 ± 0.02, *p* = 0.04. **(C)** Distribution of change in Ca^2+^-sensitivity due to troponin I phosphorylation (EC_50_ unphosphorylated/EC_50_ phosphorylated). Data from Table 5. The mean change is 0.50 ± 0.06-fold (*n* = 9), ΔpCa50 = −0.30.

What could be the underlying reason for this consistent and small effect of mutations on EC_50_? We will consider two possible mechanisms that are not necessarily mutually exclusive. Firstly, it could be that the limit is set by the capacity of the EC coupling system that supplies activating Ca^2+^ and that striated muscle cannot work in a way compatible with life outside these limits; alternatively it may be due to a fundamental property of the troponin system and the permitted conformational transitions compatible with efficient regulation.

Before attempting to discuss these mechanisms it is worthwhile considering some additional evidence on Ca^2+^-sensitivity shifts. Perhaps the most puzzling observation is that there appears to be no correlation between the Ca^2+^-sensitivity shift and disease severity. Skeletal myopathy mutations that cause life-threating muscle weakness from birth and often require mechanical assistance in breathing (Ravenscroft et al., 2015), have the same Ca^2+^-sensitivity shifts as dilated cardiomyopathy mutations which are considerably less lethal (Hershberger et al., 2013). Whilst heart muscle has compensatory strategies not available in skeletal muscle to account for this difference, the small change in Ca^2+^-sensitivity even in the most severe skeletal muscle disease might be indicative of a fundamental structure-based limit on changes in EC_50_.

Consideration of the Ca^2+^-sensitivity shifts in cardiomyopathies (Tables 1, 3) do not indicate any correlation with disease severity. Any relationship that may exist is masked by the extreme variability of Ca^2+^-sensitivity shift measurements. For instance, the “severe” TNNI3 R145G HCM/RCM-linked mutation features at both extremes of the Ca^2+^-sensitivity range (1.15x and 3.65x); for the 6 assays in the table the mean is 1.84, close to the mean of all 71 HCM measurements (1.87). The same variability can be seen with other mutations where multiple values are available: ACTC E99K, *n* = 5, 1.24–2.45 mean 1.85; TPM1 E180G, *n* = 4, 1.30–2.75, mean 1.78. The second relevant observation is that the physiological modulation of cardiac muscle myofilament Ca^2+^-sensitivity due to phosphorylation of troponin I by protein kinase A has been known to be a 2–3-fold shift for many years (Solaro et al., 2008). Table 5 lists a number of recent determinations of this Ca^2+^-sensitivity shift in several species and measured by both loaded and unloaded assays illustrating its small range. Figure 1C shows how the magnitude and distribution of measured changes is similar to the changes induced by disease-causing mutations. It would be logical to conclude that this represents the range of achievable Ca^2+^ sensitivity shifts in cardiac muscle due to the limitations of the EC coupling system.

**Table 5:** **Ca^2+^ sensitivity change due to troponin I phosphorylation 8 independent measurements of the phosphorylation-induced change of EC_50_ shown as ratio of EC_50_ unphosphorylated/phosphorylated (uP/P)**.

**EC_50_**	**wt/mutant EC_50_ ratio**	**Measured in**	**References**
Human failing/donor	0.57	IVMA	Messer, 2007; Messer et al., 2007
Human failing/donor	0.68	Human fibers	van der Velden et al., 2003
Donor uP/P	0.34	IVMA	Song et al., 2011
Donor uP/P	0.32	IVMA	Bayliss et al., 2012
Donor uP/P	0.34	IVMA	Memo et al., 2013
Mouse uP/P	0.33	IVMA	Song et al., 2010
Mouse uP/P	0.50	IVMA	Memo et al., 2013
Mouse uP/P	0.74	Myofibrils	Vikhorev et al., 2014
WT cTnI/cTnI-DD	0.69	Fibers X	Biesiadecki et al., 2007

*Measurements were made with troponin (IVMA) or skinned muscle from human (donor) or mouse heart. The mean change is 0.50 ± 0.06-fold (range 0.32–0.74)*.

In principle, it should be possible to go beyond the Ca^2+^-sensitivity limits set by EC coupling in an *in vitro* system where Ca^2+^ binding affinity can be much greater or much less than the native troponin. Cardiac troponin C presents extreme examples in a single molecule. Only site II binds Ca^2+^ in the physiologically relevant range (2.5 × 10^5^ M^−1^) and so is solely responsible for Ca^2+^-regulation (Holroyde et al., 1980). A few amino acid changes in the EF-hand motifs results in sites that do not bind Ca^2+^ (Site I) or sites that bind Ca^2+^ 200x tighter (sites III and IV) and are permanently occupied by Ca^2+^ or Mg^2+^ (Li and Hwang, 2015). Thus, it would seem that neither a very high Ca^2+^ sensitivity nor a very low one are able to participate in regulation. How much deviation of Ca^2+^ affinity from the norm is compatible with muscle regulation?

It is known that for mutations, the small Ca^2+^-sensitivity changes correlate with Ca^2+^ binding affinity to thin filaments (Robinson et al., 2007). In a study of mutations induced in skeletal muscle troponin C, Davis et al. achieved a 243-fold range of Ca^2+^ binding affinities for troponin C. However, this did not translate into such a great range when Ca^2+^-binding was measured in the presence of TnI (96-148) and caused a still smaller shift in the Ca^2+^-sensitivity of force production (Davis et al., 2004). Thus, the most extreme Ca^2+^-sensitizing mutation, V45Q increased TnC Ca^2+^ binding affinity 19-fold, but the increase was only 3.1-fold when measured in the presence of the TnI peptide and Ca^2+^-sensitivity in skinned fibers was just 2.3-fold more than wild-type. This is within the same range of many HCM-causing mutations (Table 1). A similar picture emerges from Cardiac troponin C where the single regulatory Ca^2+^-binding site simplifies the argument: V44Q increases Ca^2+^-binding affinity to TnC 6.5-fold but increases myocyte Ca^2+^-sensitivity by just 3.4-fold (Parvatiyar et al., 2010). Thus, it seems that the structure of troponin and its interactions with the rest of the thin filament does limit the consequences of a modification that increases Ca^2+^ binding affinity.

A slightly different situation arises when Ca^2+^ binding affinity is less than wild-type. Davis et al., noted that the mutations that decreased Ca^2+^ binding affinity the most (F26Q, 63-fold, I37Q, 24-fold and I62Q, 10-fold) could not properly regulate force in skinned fibers since they only produced about 13% of the maximal force of wild-type muscle at saturating Ca^2+^ concentrations. On the other hand, two less extreme mutations, M81Q and F78Q decreased Ca^2+^-sensitivity whilst retaining the same maximum force production as wild type. In these cases, again, the increased Ca^2+^ binding affinity for TnC was substantially greater than the increased Ca^2+^-sensitivity of skinned fibers (5.9x vs. 1.8x for M81Q and 8.4x vs. 4.2x for F78Q). Thus, thin filament structure seems to limit the possible effects of changes in Ca^2+^-binding affinity.

It is self-evident that changing myofilament Ca^2+^ sensitivity will affect contractile output in muscle. It is well-established that EC_50_ for skinned muscle fibers is about 1 μM and that Ca^2+^-activation of contraction is highly cooperative. Most measurements suggest a five-fold range in free Ca^2+^ concentration during a cardiac muscle contraction. Peak Ca^2+^ concentration is about 600 nM at rest and can be substantially higher during adrenergic stimulation, thus normally muscle is only partially activated (Negretti et al., 1995; Dibb et al., 2007).

Figure 2 shows a real life example: in a mouse model of HCM (ACTC E99K) we measured both the Ca^2+^-activation curve for myofibrils and the contractility of intact papillary muscle as well as the Ca^2+^-transient (Song et al., 2013). Under the conditions of this experiment the Ca^2+^ transient was the same in Wild-type and ACTC E99K muscle, Ca^2+^ sensitivity was 0.8 μM for wild-type and 0.34 μM for ACTC E99K with a Hill coefficient of about 4. The increase in Ca^2+^-sensitivity due to the ACTC E99K HCM mutation corresponds to an approximately four-fold increase in twitch force in the absence of a change in the Ca^2+^-transient that was actually observed.

**Figure 2:**
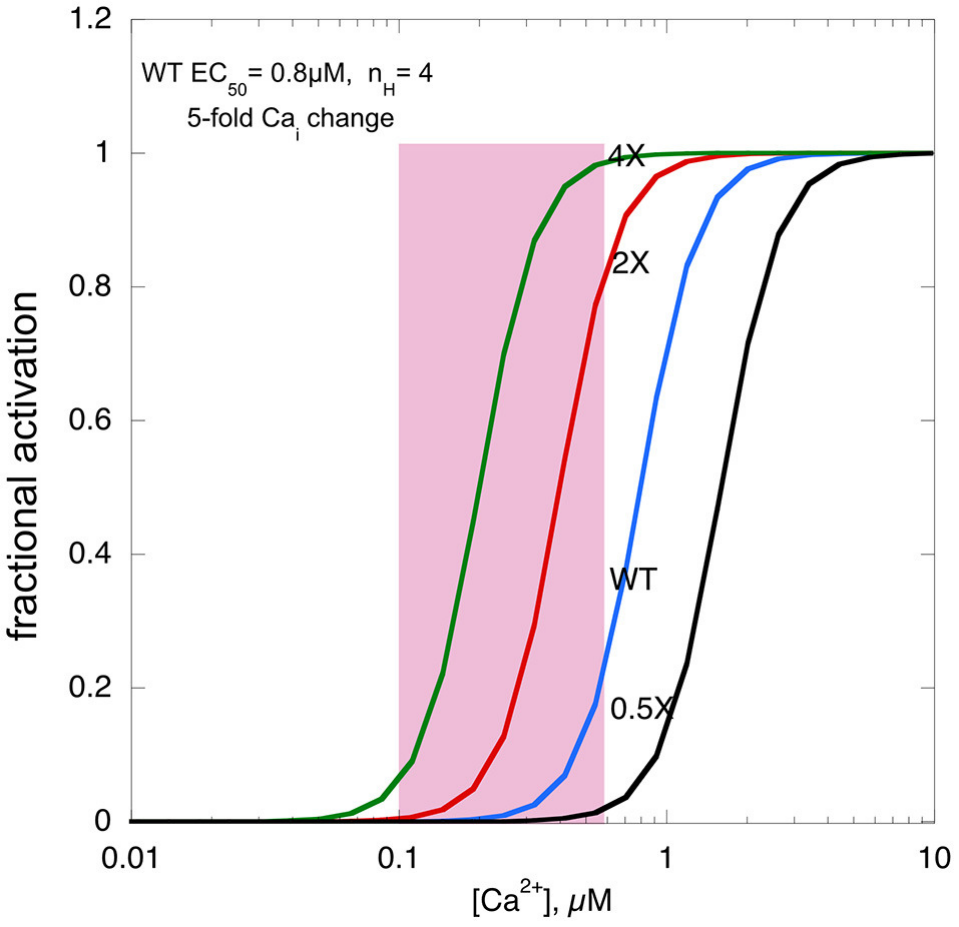
**The effects of changing Ca^2+^-sensitivity on contractility**. Ca^2+^-activation curves for mouse myofibrils with EC_50_ of 0.8 μM, a Hill coefficient of 4 and a [Ca^2+^]_i_ range from 500 nM at peak to 100 nM when relaxed (pink box). The curves with two-fold higher Ca^2+^ sensitivity, as found with HCM mutations, four-fold higher Ca^2+^ sensitivity and 0.5-fold Ca^2+^-sensitivity, as may be found in some DCM mutations, is plotted for comparison.

We can use this model to consider what would happen if Ca^2+^-sensitivity changed beyond the normal range. If myofilament Ca^2+^-sensitivity was 4 times normal, maximum force would reach close to 100%, leaving no range for it to be modulated by adrenergic agents. Moreover, it is likely that the muscle would not fully relax, since, based on the five-fold range of the Ca^2+^ transient even at the lowest Ca^2+^ level force would be 5–10%, a substantial fraction of the peak force of wild-type muscle, thus the hypercontractile phenotype would impose a major defect in relaxation, much more severe than the diastolic dysfunction associated with HCM mutations with only a 1.8-fold average Ca^2+^ sensitivity increase.

If myofilament Ca^2+^-sensitivity were decreased to half the normal, contractility would be very low indeed. The fact that mutations that decrease Ca^2+^-sensitivity are not lethal and indeed in transgenic mice, may exhibit little phenotype, is probably due to a compensatory increase in the Ca^2+^-transient (Du et al., 2007). However, this compensation may not be enough to support normal contraction in the long term, leading to DCM, the phenotype commonly associated with reduced Ca^2+^-sensitivity.

## Conclusion

The objective of this article was to confirm that Ca^2+^-sensitivity of contractility only varies within an narrow range of three-fold above and below the normal EC_50_ at rest and to investigate why this should be. The high cooperativity of muscle activation by Ca^2+^ means there is a narrow [Ca^2+^] range between relaxed and active muscle. It would appear that the excitation-contraction coupling machinery of the cell has limited ability to change the amplitude of the Ca^2+^-transient or baseline [Ca^2+^] to compensate for changes in EC_50_; thus increased Ca^2+^-sensitivity would be limited by inability to relax and reduced Ca^2+^-sensitivity would be limited by inability to contract. It is intriguing that the Ca^2+^-sensitivity range of the thin filament itself is independently limited. Mutations that change Ca^2+^-binding affinity to TnC by a large amount nevertheless only produce a small change in EC_50_ for activation of loaded or unloaded contractility *in vitro*. Whether this property is an evolutionary adaptation that limits the deleterious effects of mutations in thin filaments or simply fortuitous in unknown.

## Author contributions

The author confirms being the sole contributor of this work and approved it for publication.

## Funding

SM's research is funded by British Heart Foundation programme grant RG/11/20/29266.

### Conflict of interest statement

The author declares that the research was conducted in the absence of any commercial or financial relationships that could be construed as a potential conflict of interest.

## Abbreviations

HCM: hypertrophic cardiomyopathy
RCM: Restrictive cardiomyopathy
DCM: dilated cardiomyopathy
EC_50_: Ca^2+^ concentration that gives 50% maximal activation
pCa_50_: –log EC_50_.
